# SOMMD: an R package for the analysis of molecular dynamics simulations using self-organizing map

**DOI:** 10.1093/bioinformatics/btaf308

**Published:** 2025-05-15

**Authors:** Stefano Motta, Lara Callea, Shaziya Ismail Mulla, Hamid Davoudkhani, Laura Bonati, Alessandro Pandini

**Affiliations:** Department of Earth and Environmental Sciences, University of Milano-Bicocca, Milan, 20126, Italy; Department of Earth and Environmental Sciences, University of Milano-Bicocca, Milan, 20126, Italy; Department of Computer Science, Brunel University of London, Uxbridge UB8 3PH, United Kingdom; Department of Computer Science, Brunel University of London, Uxbridge UB8 3PH, United Kingdom; Department of Earth and Environmental Sciences, University of Milano-Bicocca, Milan, 20126, Italy; Department of Computer Science, Brunel University of London, Uxbridge UB8 3PH, United Kingdom; The Thomas Young Centre for Theory and Simulation of Materials, London SW7 2AZ, United Kingdom

## Abstract

**Motivation:**

Molecular dynamics (MD) simulations provide critical insights into biomolecular processes but they generate complex high-dimensional data that are often difficult to interpret directly. Dimensionality reduction methods like principal component analysis, time-lagged independent component analysis, and self-organizing maps (SOMs) have helped in extracting essential information on functional dynamics. However, there is a growing need for a user-friendly and flexible framework for SOM-based analyses of MD simulations. Such a framework should offer adaptable workflows, customizable options, and direct integration with a widely adopted analysis software.

**Results:**

We designed and developed SOMMD, an R package to streamline MD analysis workflows. SOMMD facilitates the interpretation of atomistic trajectories through SOMs, providing tools for each stage of the workflow, from importing a wide range of MD trajectories data types to generating enhanced visualizations. The package also includes three example projects that demonstrate how SOM can be applied in real-world scenarios, including cluster analysis, pathways mapping and transition networks reconstruction.

**Availability and implementation:**

SOMMD is available on CRAN (https://CRAN.R-project.org/package=SOMMD) and on GitHub (https://github.com/alepandini/SOMMD).

## 1 Introduction

Molecular dynamics (MD) simulations are invaluable tools to study the dynamic behavior of biomolecules, offering a detailed view of molecular processes at the atomistic level and the ability to gain insights on the interplay between conformations and functions (Dror *et al.* 2012). However, to understand these molecular processes, it is crucial to describe the conformational states sampled by the system during the simulation and their relationships (Hollingsworth and Dror 2018).

Exploring the conformational space through MD simulations can generate high dimensional data due to the number of degrees of freedom in complex molecular systems. A significant challenge lies in interpreting this large volume of data and transforming it into meaningful representations that can reveal the relationships between different states (Hollingsworth and Dror 2018). Such representations should be not only informative but also interpretable, enabling researchers to gain insights into the functional dynamics of the systems being studied. Dimensionality reduction methods, which transform high-dimensional data into lower-dimensional representations, have become indispensable in this context. Among the available approaches, methods preferentially used for simulation data include principal component analysis (PCA), time-lagged independent component analysis (TICA) (Molgedey and Schuster 1994, Noé and Nüske 2013), and self-organizing maps (SOMs) (Kohonen 1990, Shao *et al.* 2007, Fraccalvieri *et al.* 2011, Kohonen 2013, Bouvier *et al.* 2015).

SOMs provide a distinctive approach to organize complex, high-dimensional data into a two-dimensional grid of neurons, where each neuron represents a specific region in the data space. Topological relationships between these regions in the original data space are preserved in the SOM. In recent years, SOMs have been successfully used to cluster conformational ensembles (Fraccalvieri *et al.* 2011, Bouvier *et al.* 2015, Motta *et al.* 2023), and to reconstruct conformational pathways of protein (un)folding (Motta *et al.* 2021, Hendrix *et al.* 2022), protein–protein binding (Yuan *et al.* 2024a,b), and ligand binding (Motta *et al.* 2022, Tripathi and Nair 2023), with the ability to provide estimates of binding kinetics constants (Rubina and Moin 2023, Callea *et al.* 2024). Despite their potential, there is currently no user-friendly and extendable framework that allows researchers to work seamlessly in a single environment and to develop ad-hoc workflows for specialized SOM-based analyses. To address this gap, we have chosen the R environment for its versatility and extensive ecosystem of packages. Currently available packages on CRAN [e.g. Kohonen (Wehrens and Kruisselbrink 2018), aweSOM (Boelaert *et al.* 2021), popsom7 (Hamel *et al.* 2025)] are general-purpose implementations that have not been designed to process MD data and do not include functions to reconstruct conformational pathways and model transitions between states. Therefore, we have developed SOMMD, a dedicated package for the analysis of MD simulations using SOMs.

SOMMD is an R package specifically designed to meet the needs of the MD community by providing a format-agnostic tool, support for processing large datasets, flexible user-defined geometrical descriptors of dynamics, effective visualization tools, and the ability to convert trajectory data into interpretable models of conformational transitions. To this end, SOMMD offers R functions to read and process MD trajectories of various formats, calculate descriptors for SOM training, extract representative structures of the sampled states, trace pathways followed during the simulations on the trained SOM, construct transition graphs between SOM neurons, and generate customizable visualizations for effective analysis. SOMMD offers a user-friendly and integrated solution, allowing researchers to easily model and interpret the dynamics of biomolecular systems using SOMs.

The SOMMD package includes both pre-defined workflows in the form of R notebooks which serves as “scenario recipes,” as well as a set of R functions for developing ad-hoc workflows for the analysis of molecular simulations. Simulation data required to execute the workflows are hosted on Figshare (see Data availability) and can be automatically downloaded and loaded from the notebooks.

## 2 Description

**Figure 1.**
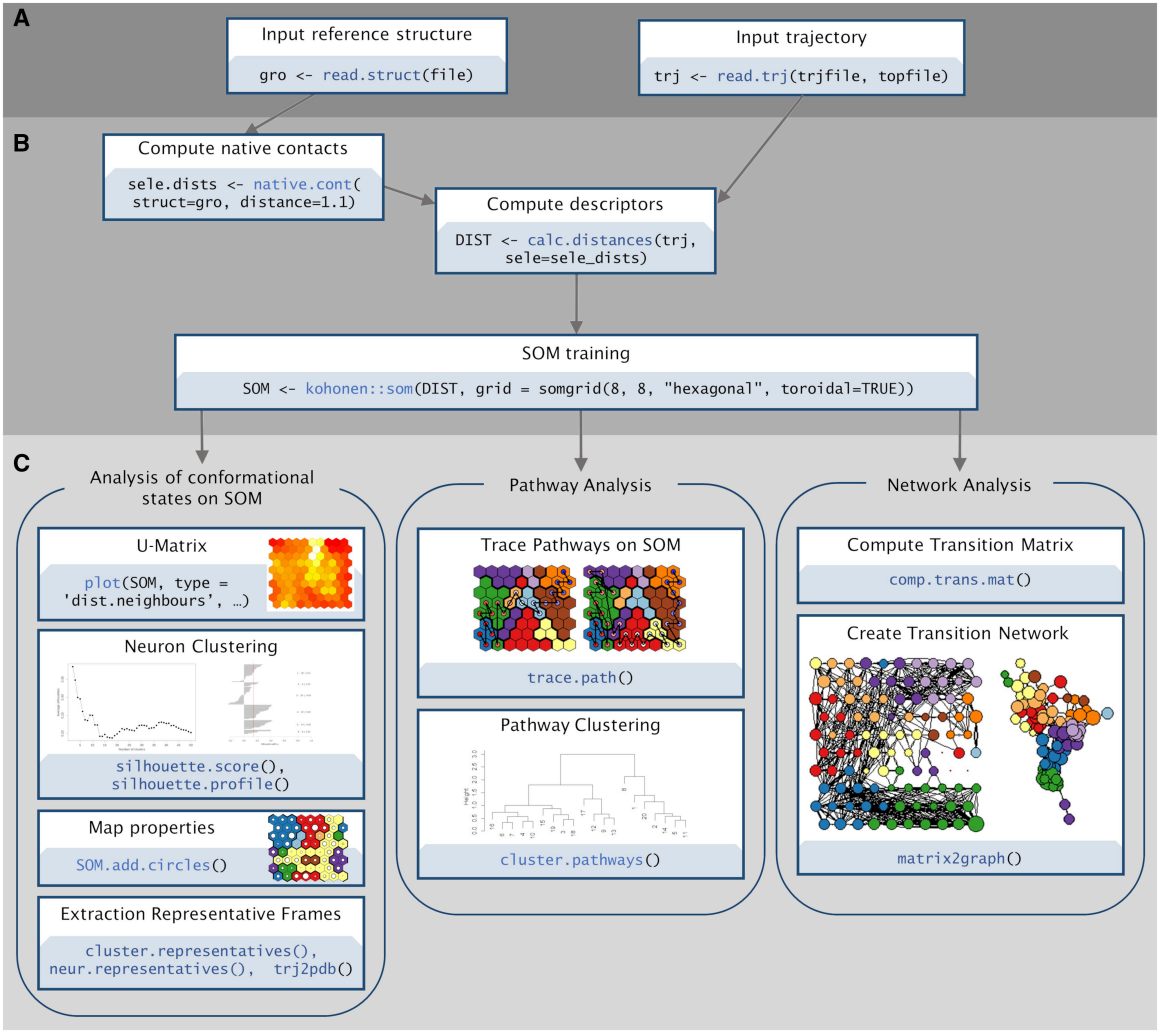
Summary diagram of the SOMMD package architecture and analysis workflow. The package provides functions to read the structure topology and trajectory files (A. *input*), to compute the desired conformational descriptors and to train the SOM (B. *preprocessing and training*). From a trained SOM analysis and visualization can be performed on three levels: conformational states, pathways, and transition networks (C. *analysis and visualization*).

The SOMMD package was designed to streamline the generation of interpretable Self-Organizing Maps from MD data in R. To this end it takes advantage of powerful data classes from bio3d (Grant *et al.* 2006) and machine learning functions from the Kohonen (Wehrens and Buydens 2007, Wehrens and Kruisselbrink 2018) and cluster (Maechler *et al.* 2022) packages, but it extends them. Specifically, SOMMD introduces a more general-purpose trajectory class and dedicated visualization functions for MD data. The architecture of SOMMD is structured into three distinct components, each fulfilling a specific role in the analysis workflow (Fig. 1):

- *input:* this initial step involves the handling and preprocessing of input data, introducing a new core class for trajectory data (“trj”). An additional class is provided to represent structural files in GROMOS format (“gro”). These two new classes are designed to complement the existing molecular structure classes in the bio3d package. This implementation ensures compatibility with widely used input formats and extends the capabilities of the bio3d package (Grant *et al.* 2006) for structural and trajectory processing.
- *preprocessing and training*: the input data are pre-processed to compute descriptors for the variables used in training the SOM. These descriptors typically consist of a subset (or the entire set) of relevant interatomic distances for a group of atoms, but the package also supports user-defined geometrical measures. The map is trained using a wrapper around functions provided by the Kohonen package (Wehrens and Buydens 2007, Wehrens and Kruisselbrink 2018).
- *analysis and visualization*: this step provides functions to analyze and identify microstates and macrostates. Time-dependent relationships between states can be reconstructed and visualized as pathways on the map and as transitions in a graph model. SOMMD also offers functions for ad-hoc mapping of time-dependent properties on both the SOM and the transition graph. Various workflows can easily be constructed to extract system-specific information tailored to the biomolecular process under investigation.

The next section presents three example scenarios that are included as R Markdown notebooks in the package. These notebooks serve as prototypical examples of the most common use cases for SOMMD.

## 3 Usage scenarios

The R package includes example notebooks that illustrate how SOM analysis can be performed on previously published and validated cases.

*Clustering of MD trajectories*: The first scenario provides a brief introduction to SOM training with SOMMD. In this case, the user is interested in describing the conformations sampled during MD simulations. The study case is a set of multiple unbiased simulations of the FOXP1 protein DNA-binding domain. By applying SOMMD, it is possible to extract representative structures of clusters and create informative plots for selected property. Additionally, sampling can be assessed by remapping multiple replicas.

*Analysis of pathways:* This scenario demonstrates how to use SOMMD to compare different replicas for a process of interest recovering alternative pathways. In the present case, the process was the unfolding of a protein domain generated through steered MD (Motta *et al.* 2021). SOMMD can be used to obtain a clustering of pathways based on the sequence of neurons sampled during the different replicas.

*Transition network analysis*: This scenario demonstrates how to build a transition matrix starting from the mapping of each frame of the simulation on the SOM. Starting from a metadynamics reconstruction of the ligand-binding process (Callea *et al.* 2021), an approximate transition matrix is built according to the observed number of transitions between pairs of neurons. The visualization of the SOM neurons as a graph provides a unified picture of the sampled pathways.

Summary descriptions of the workflows and detailed results for each scenario are reported in Sections 1–3, available as supplementary data at *Bioinformatics* online. Moreover, for users working with large datasets, Section 4, available as supplementary data at *Bioinformatics* online, outlines practical tips for managing memory and computational efficiency, ensuring that SOMMD remains scalable and effective even on standard workstations.

## 4 Conclusions

The SOMMD package is a versatile tool for the analysis of molecular conformations using unsupervised learning. It integrates dimensionality reduction and clustering of conformational states sampled during MD simulations. Additionally, through dedicated functions, it facilitates the visualization of alternative pathways in molecular processes and the construction of state graphs based on the transition matrix between pairs of neurons.

SOMMD addresses the need for a comprehensive framework to generate interpretable and informative representations of conformational states and their interrelationships. While other methods for dimensionality reduction, such as PCA and TICA, are commonly used for this scope, SOMs have the distinct advantage of preserving the topological relationships between microstates, providing a visual model for clustering data into distinct regions of the map. Section 5, available as supplementary data at *Bioinformatics* online, provides a detailed comparison between SOMs and PCA, demonstrating that SOMs offer a more intuitive and comprehensive understanding of molecular unfolding pathways with only a minimal increase in computational time.

SOMMD also supports customizable visualizations, simplifying the process to map specific properties or features onto the SOM grid. This flexibility can be valuable for researchers interested in visualizing and understanding system-specific properties from MD simulation data.

The package is designed for efficient analysis of large datasets within the limits of available RAM. Details on computational performance and strategies to mitigate memory limitations are provided in Section 4, available as supplementary data at *Bioinformatics* online. Like other methods for analyzing conformational dynamics, SOMMD is limited by the sampling in the original trajectory, as detection of transition pathways and state modeling requires sufficient sampling of critical functional events. Additionally, the package does not include an automated method for selecting the best input features, so identifying the important degrees of freedom is a prerequisite.

## Supplementary Material

btaf308_Supplementary_Data

## Data Availability

The MD trajectory data required to run the examples in SOMMD are openly available on Figshare under CC-BY licence, and the accompanying R notebooks in the package include the code to automatically download and process these datasets.
