# Supplementary material for: SOMMD: an R package for the analysis of molecular dynamics simulations using self-organizing map
Source: Bioinformatics. 2025 May 15;41(6):btaf308. doi: 10.1093/bioinformatics/btaf308 (PMC12187059; doi:10.1093/bioinformatics/btaf308)
Supplement: btaf308_Supplementary_Data [file btaf308_supplementary_data.pdf]

Bioinformatics, YYYY, 0–0
doi: 10.1093/bioinformatics/xxxxx
Advance Access Publication Date: DD Month YYYY
Manuscript Category

Subject Section

SOMMD: An R Package for the Analysis of Molecular Dynamics Simulations using Self-Organizing Map

Stefano Motta[1,*], Lara Callea[1], Shaziya Ismail Mulla[2], Hamid Davoudkhani[2], Laura Bonati[1], Alessandro Pandini[2,3,*]

[1] Department of Earth and Environmental Sciences, University of Milano-Bicocca, 20126 Milan, Italy

[2] Department of Computer Science, Brunel University of London, Uxbridge UB8 3PH, U.K.

[3] The Thomas Young Centre for Theory and Simulation of Materials, London SW7 2AZ, U.K.

*To whom correspondence should be addressed.

SOMMD was developed using the git version control system, and both the project and documentation are hosted on GitHub (https://github.com/alepandini/SOMMD). It is also available as an installable package from the Comprehensive R Archive Network (CRAN). SOMMD comprises a set of functions and classes designed for the analysis of molecular dynamics (MD) trajectories using Self-Organizing Maps (SOM). To illustrate its capabilities, three example scenarios are provided in the form of R Markdown notebooks. The following three sections present each of these scenarios, demonstrating the package's potential for analysing diverse study cases. For each scenario, a concise background on the analysed system and the accompanying MD simulation is offered. Due to the inherent non-deterministic nature of SOMs, the numerical results obtained from running the provided notebooks may show small variations compared to those presented here. However, the overall interpretation of the results should remain consistent. The size and number of simulations analysed here have been reduced compared to the original dataset, allowing the notebooks to be processed in under 10 minutes on a standard workstation. Tips and tricks to deal with large dataset are discussed in section 4, after the presentation of the notebooks. Finally, the section 5 reports a comparison SOM pathway analysis and PCA on the study case presented in the second scenario (Analysis of Pathways in protein unfolding simulations). For a more in-depth understanding, additional information can be found in previously published works on the same set of simulations.

1 Clustering of MD trajectory

Goal. This notebook serves as an introductory guide to SOM training using the SOMMD package. The objective of this scenario is to demonstrate how SOMMD can unveil the complex conformational landscape of a protein, leveraging the potential of Self-Organizing Maps (SOM) to create a graphical representation of the sampled states.

System description. The study case focuses on FOXP1, a member of the Forkhead box transcription factor family that is crucial for gene expression regulation in various biological processes. Its DNA binding domain (DBD) plays a pivotal role in specific interactions with DNA sequences, influencing the transcription of target genes.

Simulation data. The unbiased molecular dynamics simulation of FOXP1's DBD provides a dynamic perspective on its structural behaviour, revealing conformational changes in solution. The input simulations consist of a set of unbiased MD replicas performed using GROMACS 2018.1 with the amber14sb forcefield. The system was prepared following an equilibration protocol similar to the one described in (Motta et al 2021), and each replica was simulated for 200 ns.

Analysis steps. An 8x8 toroidal-shaped SOM was trained using input features computed on frames from the first replicas to illustrate the basic SOM training scheme. Two types of descriptors were considered: atomic coordinates of Cα atoms and a set of distances between all the Cβ atoms in contact (distance cutoff of 1.1 Å) in the native conformation. Figure S1 presents the resulting U-Matrix for both cases, indicating the degree of similarity for each neuron with its neighbouring neurons and providing insights into the spatial distribution of the input data. Regions with extensive patches of red neurons represent macrostates of the system. For macrostate identification, a second step of cluster analysis on the neurons can be performed. The optimal number of clusters is selected using a cluster quality index, e.g. the silhouette score, and in this case, 6 clusters are a good choice, as it represents a maximum of the score (Figure S2). Using SOMMD functions, it is possible to directly extract the representative conformation of a neuron or a specific cluster for visual inspection using a 3D visualizer program such as PyMOL or VMD (Pymol, Humphrey, 1996). Additionally, users interested in a specific property of the system can represent its per-neuron average value on the map. In this case, the average RMSD computed on the Cα atoms of the domain is reported (Figure S3).

Additional analysis. Frames from different replicas can be used to train the SOM and inspect the region of the map populated by each replica. This is particularly useful for assessing the convergence of simulations (multiple replicas of the same system should show similar per-neuron populations) or differences between simulations performed under different conditions (e.g., presence/absence of a ligand or introduction of protein mutations). An example of this type of plot is presented in Figure S4.

2 Analysis of Pathways in protein unfolding simulations

Goal. The aim of this notebook is to demonstrate the potential to recover different pathways sampled by the system in the simulations.

System description. Here we focus on the PAS-B domain of ARNT protein, a member of the basic helix-loop-helix (bHLH)-PAS family of transcription factors. The PASB domain, along with other regions within ARNT, contributes to its ability to dimerise with partners.

Simulation data. The unfolding of ARNT PAS-B domain was investigated by Steered Molecular Dynamics (SMD) simulations to provide insight into the importance of PAS domain unfolding in signal transmission mechanisms. The specifics of the SMD simulations setup, including force fields employed, simulation protocol, and system details, are reported in (Motta et al. 2021). The original set of SMD simulations included 50 replicas of 200 ns each at a constant velocity (0.2 nm ns^{-1}), for a total simulation time of 10 µs. In addition, 10 replicas of 1200 ns were also run at a lower velocity (0.02 nm ns^{-1}). A smaller dataset is analysed in this notebook.

Analysis steps. An 8 × 8 sheet-shaped SOM (without periodicity across boundaries) with a hexagonal lattice shape was trained using the set of pairwise distances between Cβ atoms as input features. Only distances between Cβ atoms closer than 1.0 nm in the native folded conformation were included. These distances are crucial to describe the conformation of the protein during the unfolding process.

The optimal number of clusters was selected according to the maximum silhouette score, here equal to 7 as shown in Figure S5. The values of distance between the Cα atoms of the C and N ends of the domain can be visualised on the trained SOM to detect regions of the map representing the folded or unfolded state. As shown in Figure S6 low values indicate neurons where the protein is folded (bottom right blue corner), while high values are typical of neurons where it is unfolded (upper left red corner).

SOMMD includes a function to trace the pathways sampled during each simulation onto the SOM. This enables the reconstruction of sampled pathways across different replicas and facilitates trajectory comparisons using clustering methodologies, as demonstrated in Figure S7. This comparative analysis uncovers insights into how the system behaves under constant pulling speeds.

Additional analysis. New simulations can be mapped on the trained SOM. In this case, the replicas carried out at lower pulling velocities were mapped onto the existing SOM to assess consistency with previous data. By comparing the distributions of distances of new data points from the SOM neuron vectors, it is possible to estimate the degree of similarity between each frame and its representative neuron on the map. Lower values indicate that frames are well represented by their associated SOM neurons, while higher values suggest a less adequate representation. In Figure S8, it is evident that the distances to the SOM vectors in the two cases are similar, despite the SOM was optimized to represent the original dataset. This suggest that the SOM model is transferable and can well capture features of the higher velocity SMD.

3 Transition network analysis in ligand-protein metadynamics simulation

Goal. This notebook illustrates pathways analysis from molecular dynamics simulations and how to build a graph representation of transitions between pairs of neurons.

System description. Data represents the pathways sampled during metadynamics simulation in the binding of the THS-020 ligand to the PAS-B domain of HIF-2α. HIF-2α is a target in cancer treatment and governs cellular responses to hypoxia (low oxygen levels) through dimerization with ARNT.

Simulation data. The study of the binding process of the THS-020 ligand to the HIF-2α PAS-B domain were done using metadynamics simulations biasing path collective variables (CVs) that describes the ligand position along the binding pathway. A total of 1.8 µs was simulated, enabling the observation of several binding/unbinding events. To gain a deeper insight into the metadynamics protocol and its analysis please refer to (Callea et al. 2021).

Analysis steps. At first, a comparison of different input features for SOM training are presented in the notebook. The first set of descriptors are the set of intermolecular distances between the ligand and the protein heavy atoms forming native contacts (distance < 6Å in the bound conformation). The second type of descriptors are a set of selected atoms, that are considered relevant to describe the binding process, i.e. 40 protein atoms and 5 ligand atoms. This second approach aims to describe essential aspects of the binding site and the ligand. In this case, a capping value was applied to distances exceeding 12Å to refine the analysis.

In both cases, a 10x10 sheet-shaped SOM was trained and the SOM was displayed, showcasing the average values of the CVs (S(x): position along the predefined path; and Z(x): the distance from the reference path) across the SOM, offering insights into the distribution of specific properties, as shown in Figure S9. From this representation, it is evident that the second approach gives a more comprehensive description of the bound states, reducing noise in the recording of unbound states and providing improved characterization of bound conformations. Indeed, it significantly reduces the area on the map representing the completely unbound state to just a few neurons confined in one corner. This improvement is due to the imposition of a capping value during distance calculation. Secondly, the bound states now occupy an entire side of the map rather than being confined to a corner. While the former approach effectively highlights persistence or breaking of native interactions, it limits the ability to capture new interactions that do not appear in the native conformation.

The mapping of pathways sampled during the simulation on the SOM reveals a continuous back and forth movement along the reaction path. This pattern, depicted in Figure S10, limits the interpretability of results. To facilitate the representation of all possible pathways connecting different states and to reveal the complexity of protein-ligand binding dynamics, a graph network was constructed utilizing information from the transition matrix. In this case, the optimal layout of the vertex was generated by means of a force-directed algorithm and the network graph was created with the Fruchterman-Reingold algorithm, showing multiple paths connecting unbound and bound states (left in Figure S11). Time-dependent properties can then be visualised also on the graph network. Specifically, in Figure S11, the plot of the CVs, S(x) and Z(x), is presented in the centre and right respectively, as previously displayed on the SOM in Figure S9b.

Additional analysis. Using the information derived from the transition matrix, it is possible to use a kinetic-like clustering technique with a community detection method (like the walktrap method from igraph (Humphrey 2006)). With this approach, it is possible to separate states based on the probability of transition between microstates (neurons), instead of grouping conformations only by their geometric similarity. This method identifies

densely connected subgraphs, called communities, within the graph. It is based on the tendency of short random walks to persist within the same community. In Figure S12, neurons are grouped using this approach helping in identifying two distinct paths connecting the unbound state with the two bound states.

4 Analysis of large dataset: tips and tricks

The SOMMD package is designed to handle large molecular dynamics (MD) datasets efficiently; however, it is important to consider the computational limitations, especially memory usage, when working with large trajectory datasets. In this section, we provide practical guidance for users working with large datasets typically encountered in modern MD workflows. The largest dataset provided in the accompanying notebooks consists of 20,000 simulation frames. For SOM training, 775 pairwise residue distances are calculated for each frame, which is comparable in magnitude to the dataset sizes and analysis cost reported in previous studies. Training an 8x8 SOM on this dataset takes approximately 45 minutes on a single core of a standard laptop. However, this training process is highly parallelizable and can be distributed across multiple cores, making the SOM approach extremely scalable. Training on the largest dataset analysed by the authors to date (Callea et al. 2024), which consists of 640,000 frames and 220 distances, while using a 20x20 map, took less than two days on a Ryzen 5 workstation with 12 cores. The time required for such calculations is generally not a limiting factor, even for large datasets with a substantial number of replicas. The major limiting factor is memory usage. There are three key stages where memory consumption can become significant when dealing with large datasets:

- **Reading the Trajectory:** The functions in the SOMMD package rely on R to read trajectory files (e.g., .xtc or .dcd), and for large trajectories, this can quickly exhaust system memory. To mitigate this, users can prefilter the trajectory retaining only the atoms relevant for the analysis. For example, if only the Cβ distance matrix is needed to train the SOM, users can extract and load only the Cβ atom data from the .xtc file into R. Additionally, after calculating distances, it is advisable to remove the trajectory object from the R environment to free up RAM.
- **Feature Calculation:** The calculation of features, such as the distance matrix, requires allocating memory for a matrix with dimensions corresponding to the number of features (e.g., distances) times the number of frames. To reduce memory usage, users can limit the number of features used for training the SOM. As a reference, in our dataset of 20,000 frames with 775 distances, the resulting matrix requires around 118.4 MB of memory. To reduce memory consumption, users may choose to focus on a subset of relevant distances or apply feature selection techniques to remove highly correlated or low-variance features.
- **SOM Training:** The memory required for SOM training is directly related to the size of the feature matrix. The most effective way to reduce memory usage during training is to decrease the size of the matrix, which can be achieved by reducing the number of features (as discussed above).

It is important to note that in all the studies published by the authors, SOMs were successfully trained on standard workstations with 12 cores and 16 GB of RAM. This demonstrates that, even for real-world study cases with large datasets, SOM training can be performed in a reasonable amount of time and with manageable computational resources.

5 Comparison of Pathway analysis in protein unfolding simulations with SOM and PCA

Here we present a comparison of SOMs and Principal Component Analysis (PCA) for the task of detecting the unfolding pathways of proteins obtained from steered MD simulations. At first sight, it may look that PCA is more straightforward to use as it does not require hyperparameter tuning, while SOM training involves setting parameters (map size, learning rate and the neighbourhood function). From previous studies by the authors, it was confirmed that the performance of SOMs in analysing MD pathways is not significantly influenced by these choices. Standard parameters were used across our previous work and the only adjustable value to carefully consider is the map size that affects the granularity of description of the microstates (neurons). In general, previously published studies have used map sizes ranging from 8x8 to 20x20.

PCA was performed using functions from the stats R package on the same dataset (i.e., the distance matrix) used for SOM training to ensure a fair comparison. The SOM-based analysis (Figure S13) provides a clear, two-dimensional representation where each neuron corresponds to a distinct conformation. This mapping facilitates an intuitive comparison of the unfolding pathways, as it enables the identification of the specific neurons (or clusters) traversed by each simulation replica, thus highlighting the differences in the unfolding process. In contrast, for PCA, the high-dimensional simulation data were projected onto the first two most informative principal components (Figure S14). In this projection, the first principal component primarily captures the progression of the unfolding event, while the second component is expected to differentiate among the unfolding pathways observed across replicas. However, because PCA extracts components based on their variance, each principal component can only explain a single, unidirectional mode of motion. In the example here presented, PC2 can be effectively used to separate data points from Replica 1, which is geometrically the most distinct, with positive PC2 values, while all the other replicas have negative, but similar PC2 values. This suggests that lower-ranking PCs are required for correct separation in the different unfolding pathways. A critical limitation of PCA is that its sequential extraction of orthogonal components can lead to a scenario where one component captures the most pronounced difference (e.g., the unique unfolding mode of Replica 1) at the expense of describing finer variations among the other replicas. In contrast, SOMs do not impose orthogonality constraints on the representation and this is not built to maximise variance. Instead, SOMs optimize the topological projection of all the relevant states on a discrete two-dimensional map. This allows SOMs to capture multiple subtle differences along the same dimension of the map, resulting in a more comprehensive and interpretable visualization of the complex unfolding dynamics that is driven by state separation, more than variance. This example highlights one of the intrinsic limitations of PCA and demonstrates the advantage of using SOMs, which maintains a more comprehensive and interpretable visualization of the complex unfolding dynamics while offering a 2D mapping of easy visualisation.

In terms of computational performance, PCA proved to be nearly 100 times faster than SOM (0.5 minutes vs 45 minutes on a single core of a ryzen 7 laptop), but it required twice the memory needed to train the SOM (1.8Gb vs 0.9Gb).

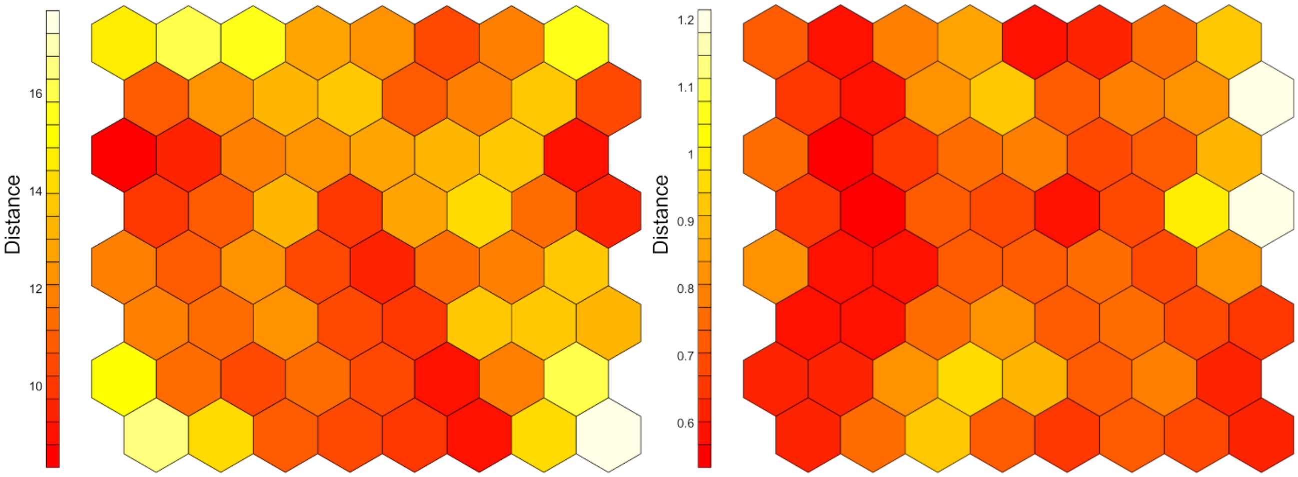

Figure S1: Neighbour distance plots (U-matrix) computed in Notebook 1 for SOM trained using as descriptors the atomic coordinates of Cα atoms (left) or a set of distances between Cβ atoms forming a contact in the native conformation (right).

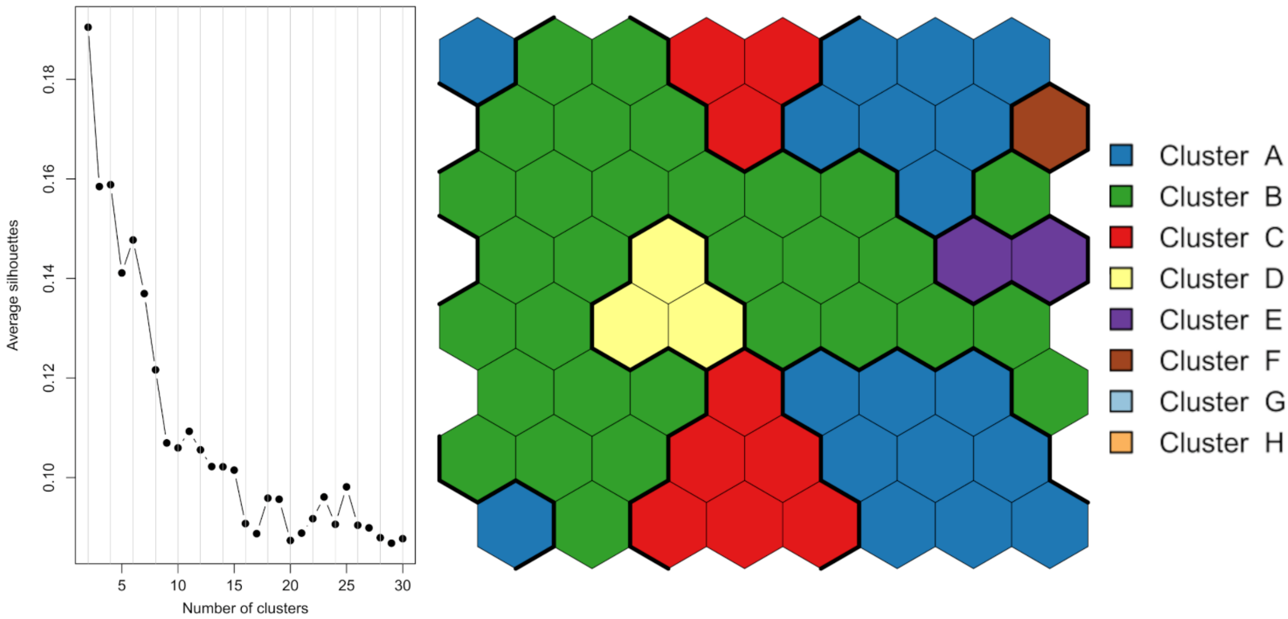

Figure S2: Cluster analysis of neurons performed in Notebook 1. Silhouette profiles (left) and SOM with neurons coloured according to the SOM clusters.

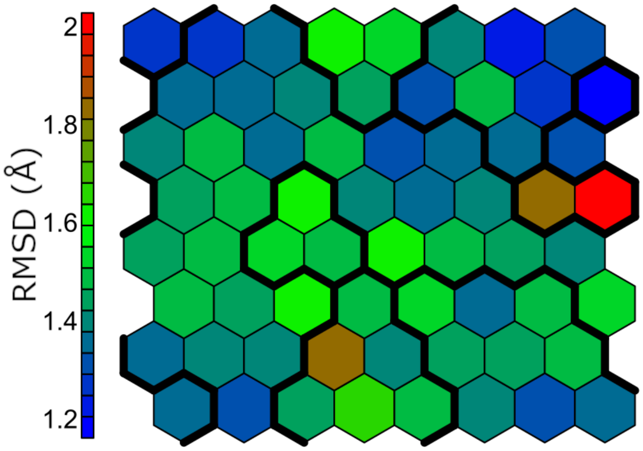

Figure S3: SOM trained in Notebook 1, with neurons coloured according to the average RMSD values computed on the Cα atoms of the domains.

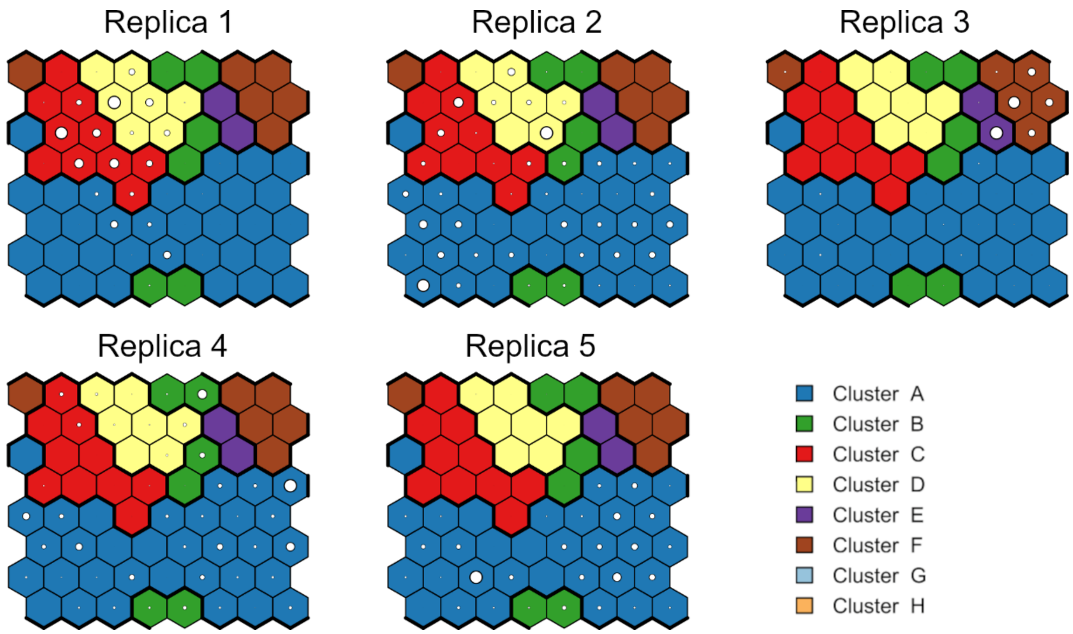

Figure S4: Per-neuron population for the SOM trained in Notebook 1 represented as circles with size proportional to the number of frames of different replicas, belonging to each neuron.

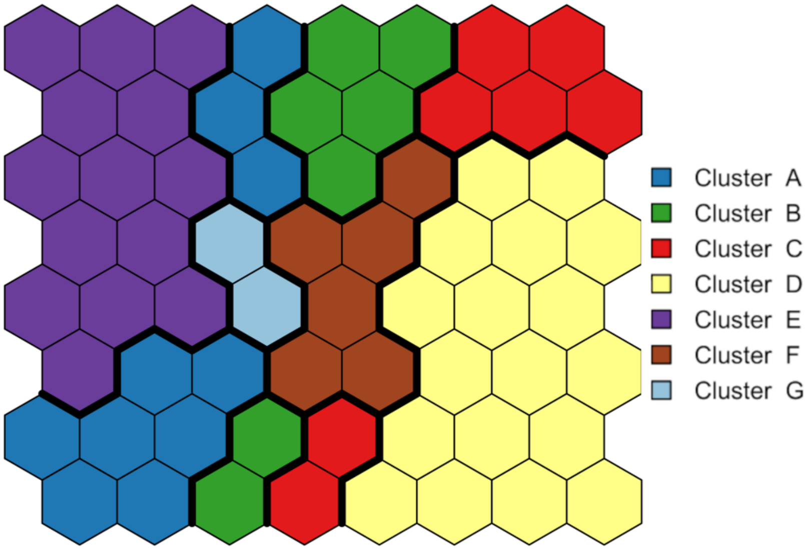

Figure S5: SOM trained in Notebook 2 with neurons coloured according to the SOM clusters.

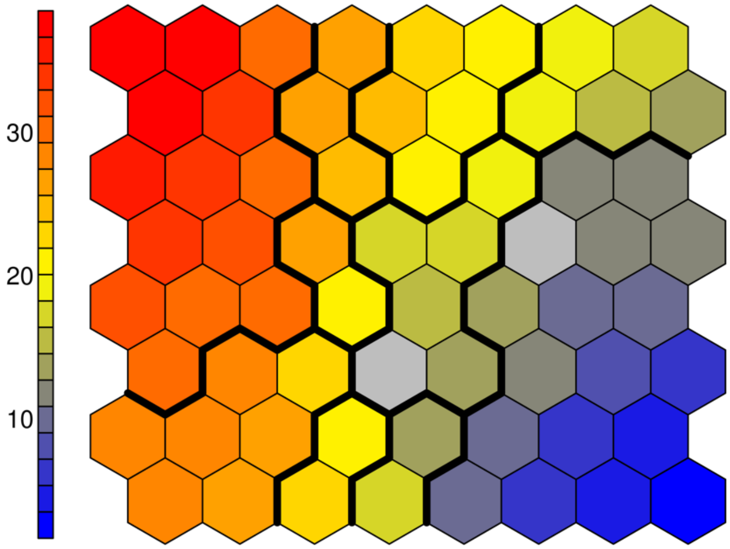

Figure S6: SOM trained in Notebook 2, with neurons coloured according to the average distance between the Cα atoms at the N-terminal and C-terminal ends of the domains.

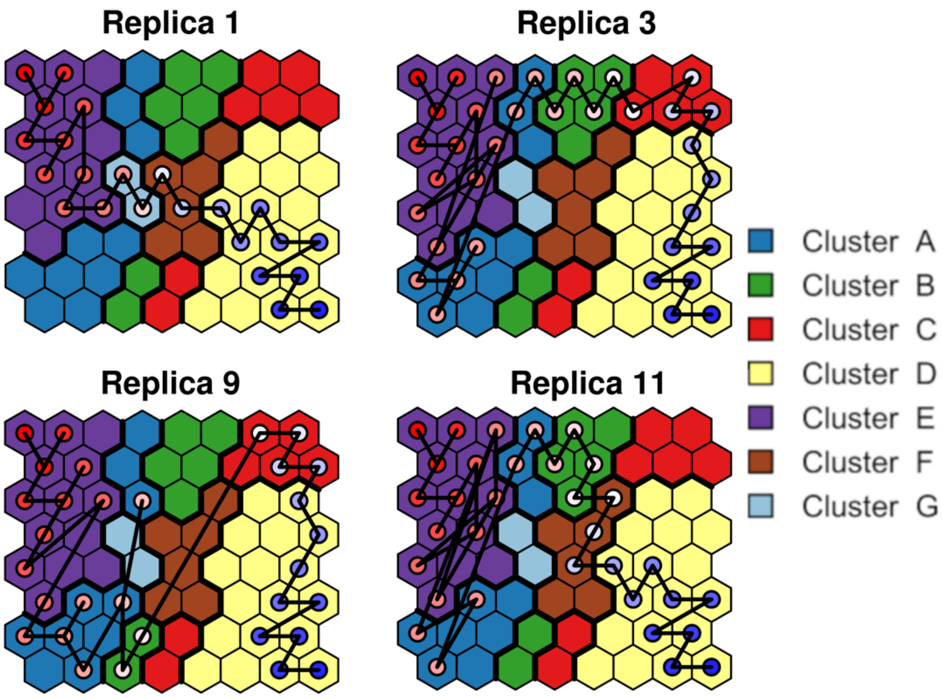

Figure S7: Tracing on the SOM of example pathways sampled in simulations analysed in Notebook 2.

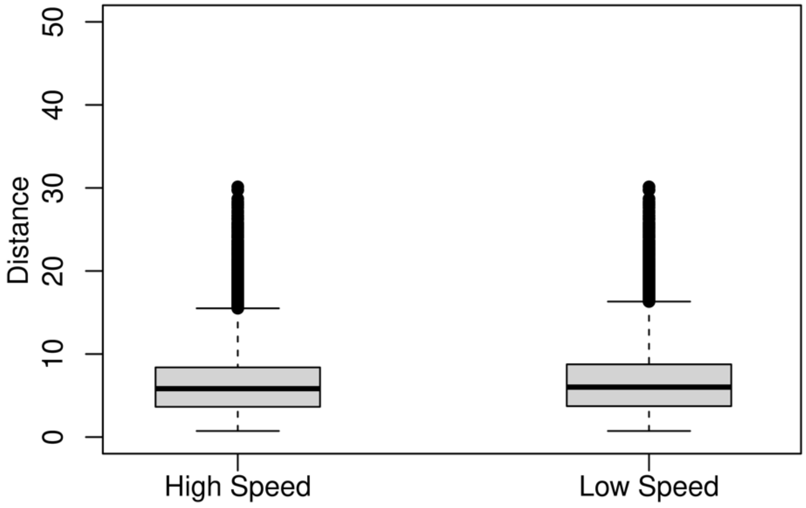

Figure S8: Boxplot reporting distances between each frame of simulations in Notebook 2 and the closest neuron on the SOM: simulations at high pulling speed (left) and at low pulling speed (right).

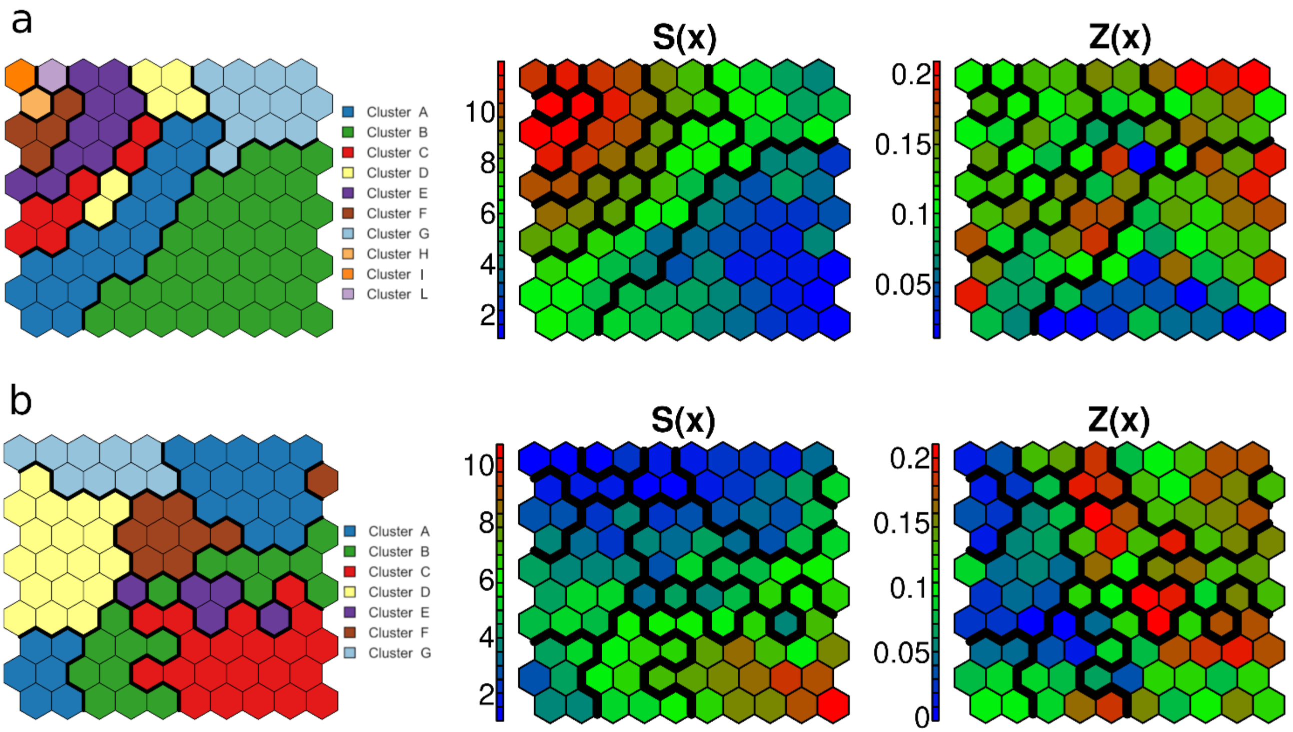

Figure S9: SOM training in Notebook 3 using different descriptors: a) The set of intermolecular distances between the heavy atoms of the ligand and the protein forming native contacts; b) The set of intermolecular distances between atoms deemed relevant for the binding process. For each set of descriptors, the SOM representing neurons coloured according to SOM clusters, the average values of the S(x) property and the average values of the Z(x) property across the SOM are reported.

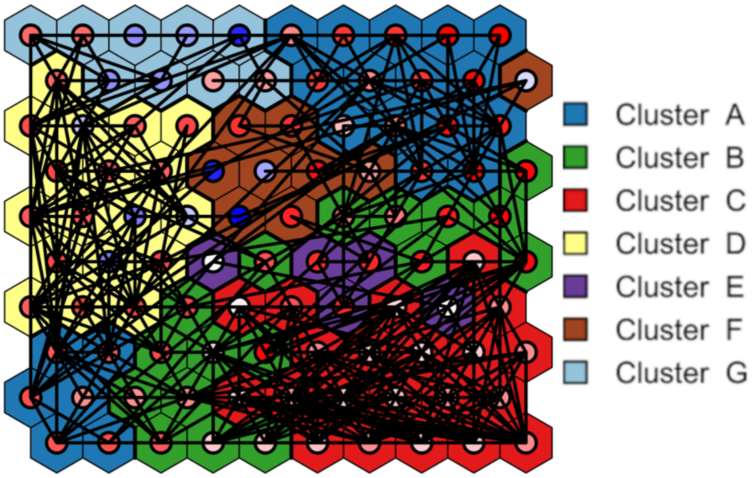

Figure S10: Tracing on the SOM of pathways sampled during the metadynamics simulation analysed in Notebook 3.

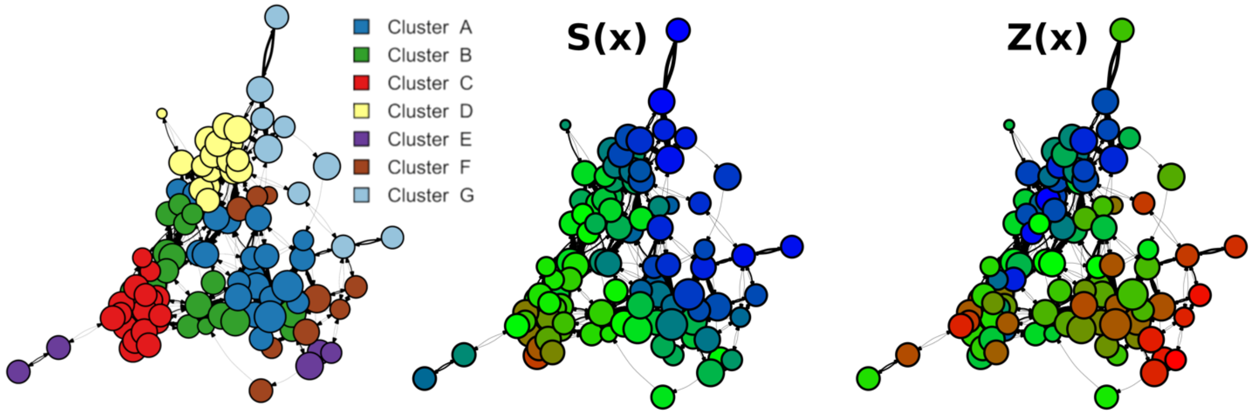

Figure S11: Transition network for the metadynamics simulation analysed in Notebook 3. From left to right, the networks are coloured according to SOM clusters, S(x) average property and Z(x) average property.

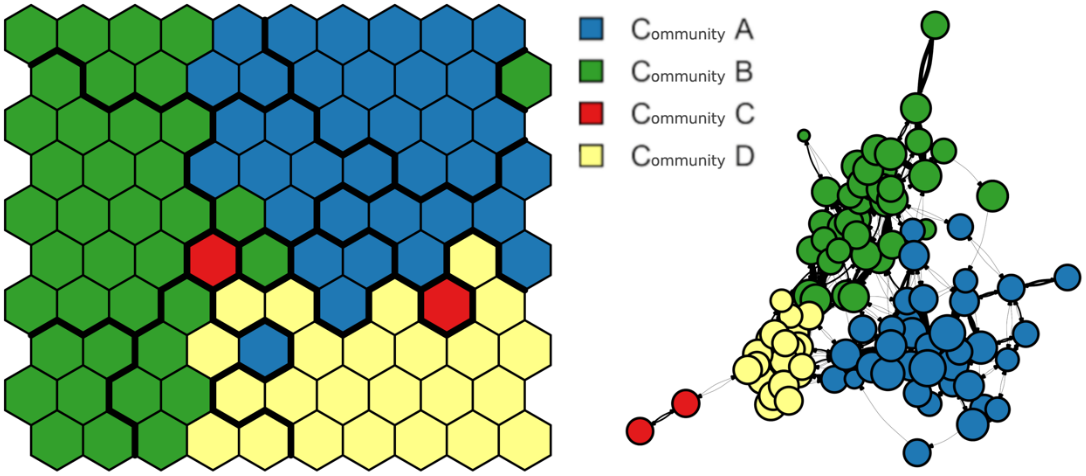

Figure S12: SOM and Network analysed in Notebook 3 with neurons coloured according to the identified communities.

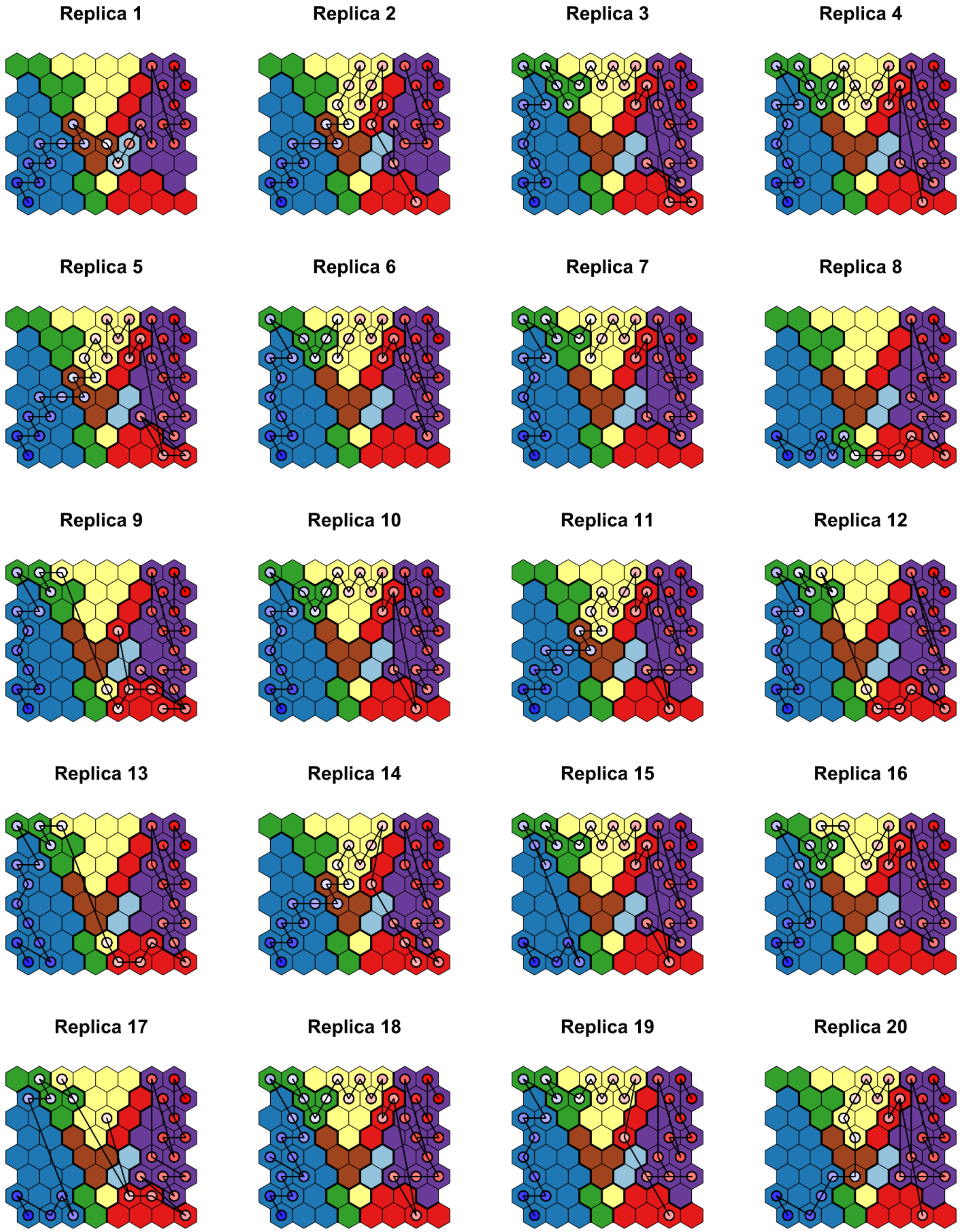

Figure S13: Tracing on the SOM of pathways sampled in the 20 replicas analysed in Notebook 2.

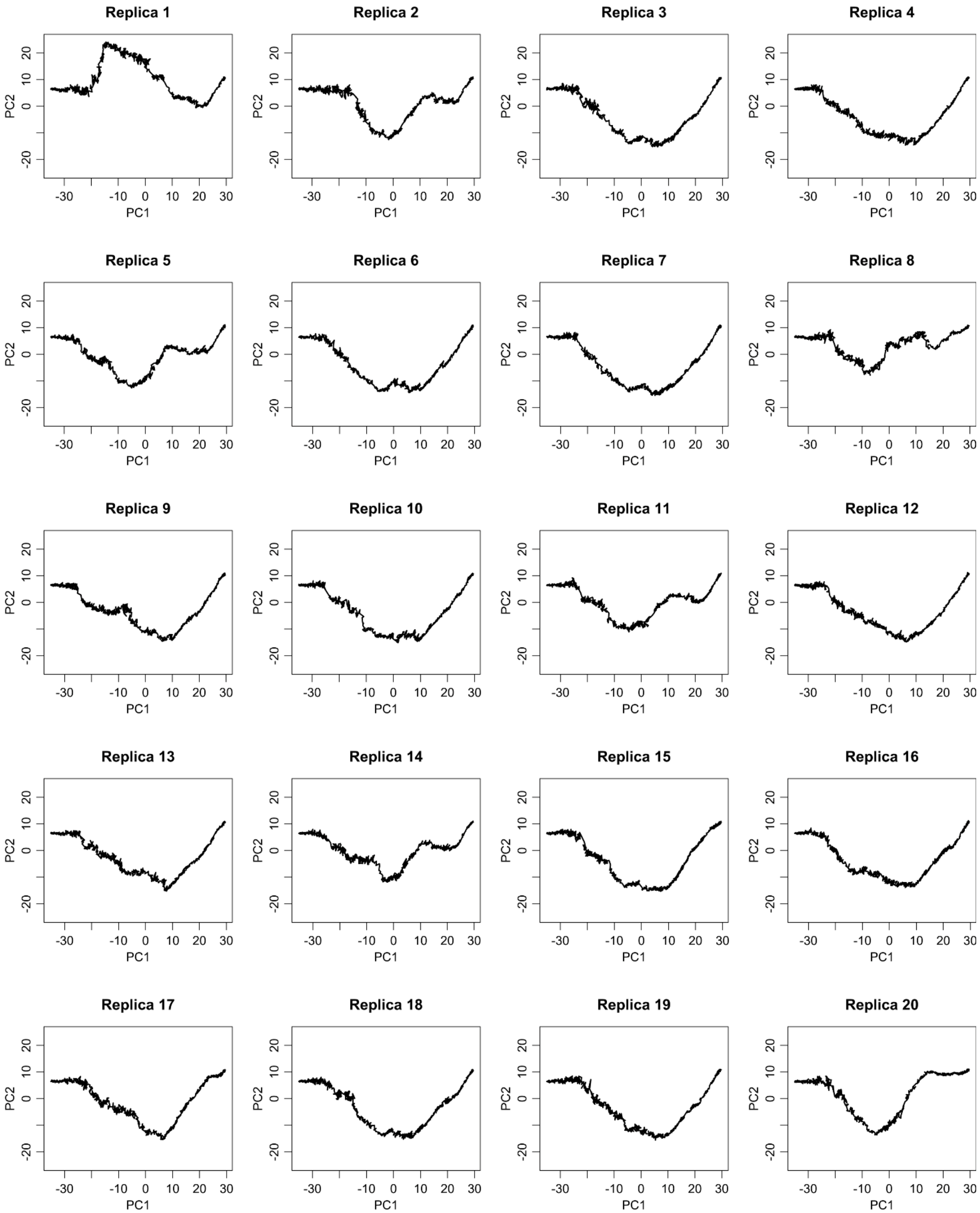

Figure S14: Plot of the first two principal components for the 20 replicas analysed in Notebook 2.

References

Callea, L., Bonati, L., & Motta, S. (2021). Metadynamics-Based Approaches for Modeling the Hypoxia-Inducible Factor 2α Ligand Binding Process. *Journal of Chemical Theory and Computation, 17*(7), 3841–3851. https://doi.org/10.1021/acs.jctc.1c00114

Callea, L., Caprai, C., Bonati, L., Giorgino, T., & Motta, S. (2024). Self-organizing maps of unbiased ligand–target binding pathways and kinetics. The Journal of Chemical Physics, 161(13). https://doi.org/10.1063/5.0225183

Csárdi, G & Nepusz T. (2006) The igraph software package for complex network research. *InterJournal Complex Systems*, 1695.

Humphrey, W., Dalke, A. & Schulten, K. (1996) VMD - Visual Molecular Dynamics, *J. Molec. Graphics*, *14*, 33-38.

Motta, S., Pandini, A., Fornili, A., & Bonati, L. (2021). Re-construction of ARNT PAS-B Unfolding Pathways by Steered Molecular Dynamics and Artificial Neural Networks. *Journal of Chemical Theory and Computation*, *17*(4), 2080–2089. https://doi.org/10.1021/acs.jctc.0c01308

The PyMOL Molecular Graphics System, Version 1.2r3pre, Schrödinger, LLC.
